# A [13]rotaxane assembled via a palladium molecular capsule

**DOI:** 10.1038/s41467-019-11635-6

**Published:** 2019-08-16

**Authors:** Jesus Ferrando-Soria, Antonio Fernandez, Deepak Asthana, Selina Nawaz, Iñigo J. Vitorica-Yrezabal, George F. S. Whitehead, Christopher A. Muryn, Floriana Tuna, Grigore A. Timco, Neil D. Burton, Richard E. P. Winpenny

**Affiliations:** 1 School of Chemistry and Photon Science Institute, The University of Manchester, Oxford Road, Manchester, M13 9PL UK; 2 Departamento de Química Inorgánica, Instituto de Ciencia Molecular (ICMol), Universidad de Valencia, 46980 Valencia, Paterna Spain; 3 Chemistry Department, Sir David Davies Building, Loughborough University, Loughborough, LE11 3TU UK

**Keywords:** Molecular capsules, Interlocked molecules

## Abstract

Molecules that are the size of small proteins are difficult to make. The most frequently examined route is via self-assembly, and one particular approach involves molecular nanocapsules, where ligands are designed that will enforce the formation of specific polyhedra of metals within the core of the structure. Here we show that this approach can be combined with mechanically interlocking molecules to produce nanocapsules that are decorated on their exterior. This could be a general route to very large molecules, and is exemplified here by the synthesis and structural characterization of a [13]rotaxane, containing 150 metal centres. Small angle X-ray scattering combined with atomistic molecular dynamics simulations demonstrate the compound is intact in solution.

## Abstract (web summary)

Mechanically interlocked molecules and molecular cages are two important themes in supramolecular chemistry. Here, the authors combine these concepts to construct a giant [13]rotaxane built around a palladium capsule, one of the most complex metallosupramolecular assemblies yet.

## Introduction

The beautiful nanoscopic molecular capsules developed by Fujita and others have frequently been studied as they allow controlled encapsulation of nanoparticles, or can act as reaction vessels for specific organic reactions^1–19^. Much of this work has involved using the interior of the nanocapsules, for example, to grow silica nanoparticles^20^. Often the bridging organic ligands are based on extended bipyridyl ligands that are modified so that additional functional groups point into the interior of the capsule, hence performing new chemistry in this very restricted space^21–23^. Other molecular capsules have been made using polyoxometallates (POM)^24^; this approach was pioneered by Müller et al.^25^, and has generated molecules with as many as 368 metal centres. While there are clearly repeat units present in these POM nanocapsules, the design principles are less immediately obvious than in the supramolecular nanocapsules involving organic bridging ligands^26–28^. There are also some examples of nanocapsules and rings involving interlocked molecules, e.g. [7]catenanes^29,30^ and large rotaxanes^31^.

We are interested in routes to bring together multiple large molecules into still larger arrays^32,33^, and therefore we are pursuing the idea that we can use a nanocapsule as the nucleation core of very large supramolecules. This is achieved by decorating the exterior of a supramolecular nanocapsule by functionalization of the organic bridging ligands. In previous work extended bipyridyl head-groups were covalently attached to the metal cage, and then palladium cations were added to make the molecular capsule^32^. Here we report a more elegant approach, where the bipyridyl head-groups are synthesized as part of a thread of a hybrid organic–inorganic [2]rotaxane^34,35^, and then the [2]rotaxanes are assembled around the Pd capsule. The result is a [13]rotaxane which has been crystallised and the structure determined. This is one of the largest structurally characterized rotaxanes reported^36–43^.

## Results

### Synthesis and structural study

**Fig. 1:**
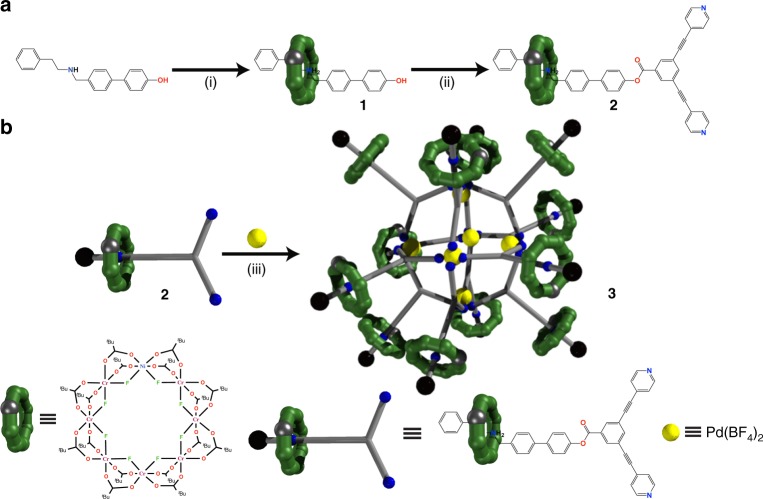
Synthetic route leading to **2** and **3**. **a** Reaction conditions: (i) CrF_3_·4H_2_O and [Ni_2_(*μ*-OH_2_)(O_2_C^*t*^Bu)_4_(HO_2_C^*t*^Bu)_4_], ^*t*^BuCO_2_H (150 °C, 24 h); (ii) 3,5-bis(pyridin-4-ylethynyl)benzoic acid, dicyclohexylcarbodiimide and 4-(dimethylamino)pyridine, THF (r.t., 48 h, Ar) Me_2_CO (50 °C, 1 h). **b** Reaction conditions: (iii) [^*n*^PrNH_3_][Cr_7_Ni(*µ*-F)_8_(O_2_C^*t*^Bu)_16_] in acetone (50 °C, 12 h)

The key component is a hybrid organic-inorganic [2]rotaxane, which is made by reaction of hydrated chromium fluoride with [Ni_2_(*μ*-OH_2_)(O_2_C^*t*^Bu)_4_(HO_2_C^*t*^Bu)_4_]^44^ using pivalic acid as a solvent (Fig. 1a) in the presence of (4′-phenethylamino)methyl-1,1′-biphenyl-_4_-ol (**A**) (6:2:1 molar ratio), which acts as a template for formation of the ring and forms the organic thread terminated by a phenol [H**A**{Cr_7_Ni(*μ*-F)_8_(O_2_C^*t*^Bu)_16_}] (**1**). A Steglich esterification reaction between 3,5-bis(pyridin-_4_-ylethynyl)benzoic acid and **1** gives [H**B**{Cr_7_Ni(*μ*-F)_8_(O_2_C^*t*^Bu)_16_}] (**2**) which is a [2]rotaxane with widely spaced pyridines with a predefined Y-type geometry^45^.

**Fig. 2:**
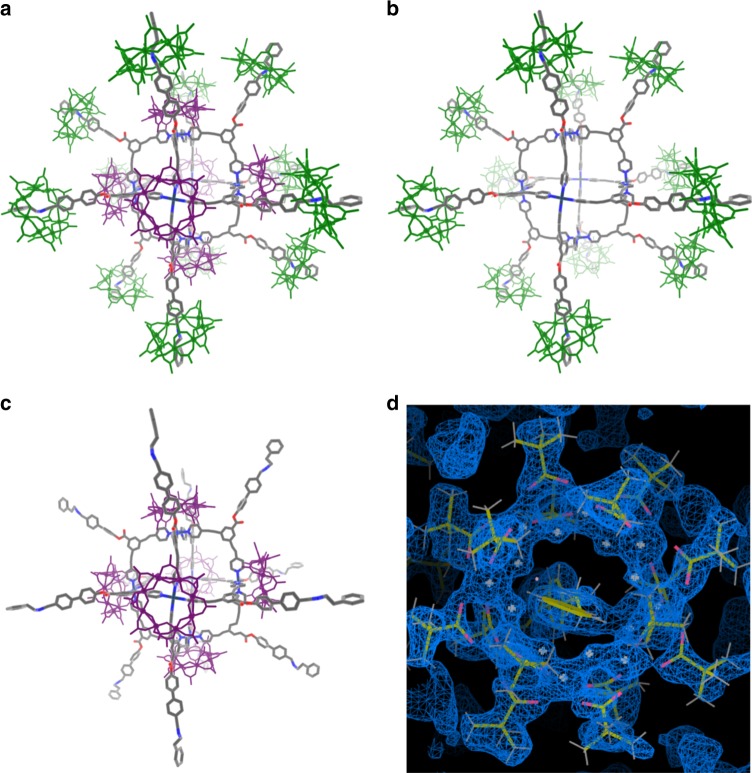
The structure of **3**. **a** The [13]rotaxane and anionic {Cr_7_Ni} rings, the {Cr_7_Ni} rings within the rotaxane shown in green, and the anionic {Cr_7_Ni} rings shown in purple. **b** The [13]rotaxane core omitting the anionic rings. **c** The {Pd_6_} core with organic threads of the [13]rotaxane, with anionic rings but omitting the {Cr_7_Ni} rings from the rotaxane. **d** 2*F*_o_−*F*_c_ electron density map and model representation showing one of the peripheral rings (map level 1.95*e*/Å^3^). (H atoms and the *tert*-butyl substituents from the pivalate groups are omitted for clarity.) Colour code for **a**–**c**: {Cr_7_Ni-Py_2_}, green; [Cr_7_NiF_8_(O_2_C^*t*^Bu)_16_]^–^, purple; Pd, dark blue. Atoms in organic threads: N, blue; O, red; C, grey

Compound **2** was reacted with Pd(BF_4_)_2_. 4MeCN; the initial study gave us {[Pd_6_[H**B**{Cr_7_Ni(*μ*-F)_8_(O_2_C^*t*^Bu)_16_}]_12_][[Cr_7_Ni(*µ*-F)_8_(O_2_C^*t*^Bu)_16_]_6_(BF_4_)_6_]} (**3**) (Fig. 2a) in very low yield, which contains both the [2]rotaxane **2** and a dethreaded ring. To improve the synthesis of **3**, the complex [^*n*^PrNH_3_][Cr_7_Ni(*µ*-F)_8_(O_2_C^*t*^Bu)_16_]^46^ was added as this can release the primary ammonium cation and then act as a bulky anion (Fig. 1b). Structural analysis showed the crystals contain a hybrid organic-inorganic [13]rotaxane **3**, where units of **2** act as linkers between the Pd^2+^ ions through the nitrogen-bipyridyl units to give a distorted octahedron of Pd sites with an internal void of *ca*. 2.3 nm (Fig. 2a, b and Supplementary Fig. 7). The Pd…Pd distances in the octahedron are between 16.224(5) and 16.275(6) Å.

Each Pd site is bound to four N-donors and has an almost square-planar geometry [Pd(1)–N = 2.017(4) Å; Pd(2)–N = 2.107(6) Å and Pd(3)–N = 1.899(5)–2.202(4) Å]. [Cr_7_NiF_8_(O_2_C^*t*^Bu)_16_]^–^ anions are found above each of the six Pd sites in the capsule (Fig. 2c), with the closest contacts being between the pivalates groups from the anion and the centre of the pyridine ring from the thread (average distance 2.3963(6) Å). Metal ligand bond lengths fall into the ranges expected; however, the size of the structure means the structure has a lower resolution than smaller molecules. An electron density map at a level of 1.95e Å^−3^ is shown in Fig. 2d to demonstrate what is seen for one of the rings within the rotaxane. Notably the electron density found at the centre of the ring for these rings is absent from the rings that are charge balancing (Supplementary Fig. 5b).

Charge neutrality is partly achieved with the six ring anions that are above each Pd^2+^ ion (Fig. 2c, Supplementary Fig. 7) and there are also six tetrafluoroborate anions present. They were not located in the crystal lattice due to the enormous size of the cation and the limited resolution of the data; inclusion of six of these anions fits the elemental analysis. The complete structure therefore contains 18 {Cr_7_Ni} units; 12 within the [13]rotaxane (Fig. 2b), and 6 as anions (Fig. 2c)

Compound **3** was not the expected product from the reaction of **2** with Pd^2+^ ions. The two widely spaced pyridine groups in **2** were expected to produce a {Pd_12_} cuboctahedron^3^, which would be at the core of a [25]rotaxane. The key parameter is the bend angle between the two-pyridines of the thread of the [2]rotaxane; a {Pd_6_} octahedron should require this bend angle^47^ to be around 90°. Here we find the bend angle to be 122.12(3)°. Clearly the formation of **3** has required some adjustment of the bridging ligands.

### Small-angle X-ray scattering in solution

**Fig. 3:**
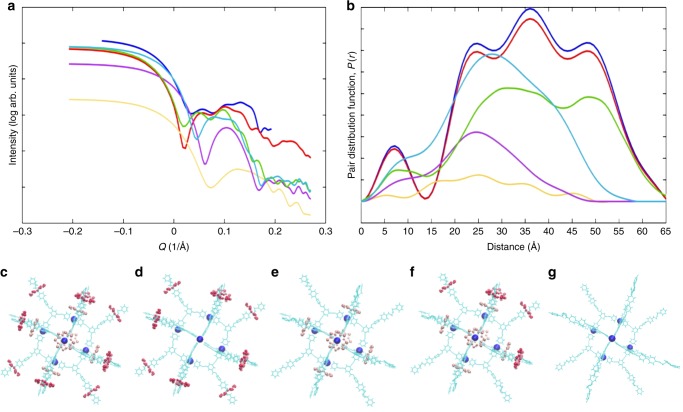
Small-angle X-ray scattering (SAXS) for **3** dissolved in THF and models to interpret the SAXS. **a** SAXS data recorded as a 25 mM solution in THF at 298 K. **b** shows a pair distance distribution function. In **a** and **b** the experimental result is shown in blue. The calculated traces are: the whole structure of **3** (red, shown in **c**); the structure lacking the six ring anions (green, shown in **d**); the structure lacking the rings on the thread of the [2]rotaxanes (purple, shown in **e**); the structure lacking half the rings from the rotaxanes (cyan blue shown in **f**); the {Pd_6_} core and organic threads only (orange, shown in **g**)

Given we were expecting a much larger cage, we have studied **3** in solution using small-angle X-ray scattering (SAXS). This has previously been used to study supramolecular aggregates of porphyrins^48,49^ and smaller hybrid rotaxanes^33^. The SAXS data unambiguously support formation of a particle with a radius of 27.4 Å in solution (Fig. 3a), which matches well with the structure of **3** in the crystal; the expected Pd_12_L_24_ supramolecule would have a radius of 50 Å^3^.

The SAXS study suggests that the structure of **3** is not an artefact caused by crystallization but is maintained in solution. However, we wished to study its formation and stability further and therefore performed all-atom simulations of the [13]rotaxane in a THF solution using GROMACS 5.1.4 molecular dynamics package^50,51^. The initial model was based on the experimental crystal structure Cartesian coordinates for the [13]rotaxane and was parameterized using the AMBER95 forcefield, as implemented in GROMACS, augmented by parameters consistent with the General Amber Forcefield^52^. Additional harmonic restraints were added to maintain the integrity of the Pd cage and [Cr_7_NiF_8_(O_2_C^*t*^Bu)_16_] rings during the simulation. The simulation began with a single molecule of **3** in a solution of 9356 THF molecules at a density of 890 kg m^−3^ in a cubic box size of 11.00 nm^3^ with six BF_4_^−^ ions to neutralize the charge.

To prepare the system prior to the simulation production runs, the system was minimized with the conjugate gradient algorithm. The minimized structure was run for 10 ns using an NPT ensemble (*T* = 298 K and *p* = 1.01325 bar) using the Nosé–Hoover thermostat^53,54^ and the Parrinello–Rahman barostat^55^. Periodic boundary conditions were used and the time step for integration was fixed at 1 fs with the neighbour list updated every 10 fs. The particle-mesh Ewald method^56^ was used to evaluate the electrostatics.

The resulting structure was then used to calculate the SAXS data and corresponding pair distribution function *P*(*r*), and the results demonstrate that the entire structure appears to be stable in solution. We compared the experimental SAXS data (Fig. 3, left) and pair distribution function *P*(*r*) (PDF) (Fig. 3, right) (shown in blue in Fig. 3) with a calculation based on the entire structure (red in Fig. 3; structure in Fig. 3c) and then examined how SAXS and PDF would vary through removing parts of the structure. Firstly, we removed the weakly bound ring anions (i.e. the structure shown in Fig. 3d); this gives the results shown in purple in Fig. 3a, b. Secondly, we removed the 12 {Cr_7_Ni} rings that are part of the [2]rotaxanes from the structure, giving the predicted trace shown as green (Fig. 3 structure shown in Fig. 3e). We also removed half of these rings, making an asymmetric compound (cyan blue in Fig. 3, structure shown in Fig. 3f) and looked at the just the {Pd_6_} core and organic ligands (orange in Fig. 3, structure shown in Fig. 3g). The best agreement is found for the complete structure and the agreement here is remarkably good. The other calculations are not consistent with the experimental observations.

### Electron paramagnetic resonance spectroscopy

We have studied the magnetism and electron paramagnetic resonance (EPR) spectroscopy of **3** (Supplementary Figs. 8 and 9) as {Cr_7_Ni} rings have been proposed as qubits for quantum information processing (QIP)^57,58^. It is likely that in any useful device a very large number of such qubits would need to be brought together to perform useful algorithms and therefore understanding the interactions between individual rings in a large supramolecule is useful. The magnetic susceptibility can be fitted using parameters for isolated {Cr_7_Ni} rings^59^, with no evidence of inter-ring interactions. Similarly, X-band continuous wave EPR spectra recorded on a frozen solution of **3** show the characteristic signals of isolated {Cr_7_Ni} rings and can be simulated with the known *g*-value for the isolated unit, without including any exchange interaction^59^.

Pulsed EPR measurements at the resonance corresponding to the {Cr_7_Ni} rings (Supplementary Figs. 10–12, Supplementary Table 2) provide a phase memory time of 425 ns, which is shorter but of the same order of magnitude as that of an isolated {Cr_7_Ni} ring^59^. This small reduction in the phase memory time is positive, in that it shows that multi-qubit arrays can be brought together without the electron spin on one sub-unit producing a catastrophic effect on the spin coherence of the another. However, unlike for [3]rotaxanes^57^, we have been unable to perform double electron-electron resonance (DEER) spectroscopy to examine how the spin on one potential qubit communicates to another. This may be due to the complexity of the structure of **3**, with multiple different qubit….qubit distances and interactions. This is a problem but also offers some potential in that if we could orientate an array of molecules of **3** we may be able to selectively address specific qubit–qubit interactions and not others. This is immensely ambitious but achievable if we can make diamagnetic equivalents of **3**; we are developing diamagnetic hybrid rotaxanes for that end^60^, but have not yet achieved assemblies as complex as **3**.

The formation of **3** leaves an intriguing question, which is why the expected {Pd_12_} cuboctahedron did not form, rather the capsule is based on a {Pd_6_} octahedron. Answering this question will require considerable further synthesis and modelling. It is particularly surprising as the presence of the {Cr_7_Ni} ring makes **2** a very bulky “bis-pyridyl” ligand, and yet the smaller, and presumably more crowded capsule results. A further target is to make a thread that will allow formation of a [3]rotaxane and then bring together a capsule where each Pd…Pd edge is bridged by a [3]rotaxane. The synthetic methods developed here and the interpretation of the SAXS data will allow us to make and characterize still larger rotaxanes.

## Methods

### Synthetic details

Unless stated otherwise, all reagents and solvents were purchased from Aldrich Chemicals and used without further purification. The syntheses of the hybrid organic–inorganic rotaxanes were carried out in Erlenmeyer Teflon® FEP flasks supplied by Fisher. Dry tetrahydrofuran was obtained by passing the solvent (HPLC grade) through an activated alumina column on a PureSolvTM solvent purification system (InnovativeTechnologies Inc., MA). Dry 1,4-dioxane was purchased from Acros Organics. Column chromatography was carried out using Silica 60 A (particle size 35–70 μm, Fisher, UK) as the stationary phase, and TLC was performed on pre-coated silica gel plates (0.25-mm-thick, 60 F_254_; Merck, Germany). NMR spectra were recorded on Bruker AV 300 and Bruker AV400 instruments. Chemical shifts are reported in parts per million (ppm) from low to high frequency and referenced to the residual solvent resonance. Elemental analyses were performed by departmental services at The University of Manchester. Carbon, nitrogen and hydrogen analysis was performed using a Flash 200 elemental analyser. Metal analysis was performed by Thermo iCap 6300 Inductively Coupled Plasma Optical Emission Spectroscopy (ICP-OES) on solid samples. [^*n*^PrNH_3_][Cr_7_Ni(*µ*-F)_8_(O_2_C^*t*^Bu)_16_] was synthesized according to methods documented in the literature^46^. The organic compounds (4′-phenethylamino)methyl-1,1′-biphenyl-4-ol (**A**) and 3,5-bis(pyridin-4-ylethynyl)benzoic acid were made by literature procedures^45^.

[HA{Cr_7_Ni(*μ*-F)_8_(O_2_C^*t*^Bu)_16_}] (**1**): Pivalic acid (20.0 g, 195 mmol), (4′-phenethylamino)methyl-1,1′-biphenyl-4-ol (0.5 g, 1.7 mmol), and CrF_3_·4H_2_O (2.1 g, 12 mmol) were heated at 140 °C with stirring in a Teflon flask for 0.5 h, then [Ni_2_(H_2_O)(O_2_CCMe_2_)_4_(HO_2_CCMe_2_)_4_] (1.2 g, 2.5 mmol) was added. After 1 h the temperature of the reaction was increased to 160 °C for 24 h. The flask was cooled to room temperature, and then acetonitrile (35 mL) was added while stirring. The green microcrystalline product was collected by filtration, washed with a large quantity of acetonitrile (200 mL), dried in air, washed with acetone and toluene, and then the remaining solid was extracted with THF to afford the desired product **1** as a green crystalline solid (1.5 g) in 35% yield. X-ray quality crystals were obtained for **1** by recrystallization from THF/MeCN. ESI-MS (sample dissolved in THF, run in MeOH): *m*/*z* = 2474 [M + H]^+^; 2497 [M + Na]^+^. Elemental analysis (% calcd., % found for C_101_H_166_Cr_7_F_8_NNiO_33_): C (48.58, 48.98), H (6.70, 6.71), Cr (14.58, 14.71), N (0.56, 0.49), Ni (2.35, 2.02).

[HB{Cr_7_Ni(*μ*-F)_8_(O_2_C^*t*^Bu)_16_}] (**2**): A mixture of **1** (1.20 g, 0.5 mmol), 3,5-bis(pyridin-4-ylethynyl)benzoic acid (0.16 g, 0.5 mmol), dicyclohexylcarbodiimide (0.10 g, 0.5 mmol) and 4-(dimethylamino)pyridine (0.06 g, 0.5 mmol) in THF (10 mL) was stirred for 2 days at room temperature under an argon atmosphere. The reaction mixture was filtered to remove the dicyclohexylurea formed and the solvent evaporated under reduced pressure. The resulting residue was purified by column chromatography. First toluene, followed by 60:1 toluene:ethyl acetate was used, which allowed **1** to be eluted, leaving the products of the reaction at the top of the column. Thereafter 10:1 toluene:ethyl acetate was used, eluting **2**. The solvent was removed under reduced pressure. Crystals of **2** were obtained from recrystallization from THF/MeCN, but they do not diffract well enough to give a high-resolution structure (0.48 g, 40%). ESI-MS (sample dissolved in THF, run in MeOH): (*m*/*z*) = 2804 [M + H]^+^; 2826 [M + Na]^+^. Elemental analysis (% calcd., % found for C_122_H_178_Cr_7_F_8_N_3_NiO_34_): C (52.26, 52.51), H (6.36, 6.39), Cr (12.98, 13.32), N (1.50, 1.66), Ni (2.09, 2.01).

[Pd_6_[HB{Cr_7_Ni(*μ*-F)_8_(O_2_C^*t*^Bu)_16_}]_12_][[Cr_7_Ni(*µ*-F)_8_(O_2_C^*t*^Bu)_16_]_6_(BF_4_)_6_]} (**3**): Compound **2** (112.20 mg, 0.04 mmol) and [^*n*^PrNH_3_][Cr_7_Ni(*µ*-F)_8_(O_2_C^*t*^Bu)_16_] (45.05 mg, 0.02 mmol) were dissolved in hot acetone (10 mL). A solution of Pd(BF_4_)_2_. 4CH_3_CN (8.88 mg, 0.02 mmol) in acetone (3 mL) was added dropwise and the green solution was stirred at 50 °C for 12 h. The solvent was removed under reduced pressure and X-ray quality crystals were obtained for **3** by recrystallization from THF/MeCN (3:1) (151.71 mg, 60 % isolated yield). A sample was stored in a desiccator for 2 days prior to submission for elemental analysis. The calculated elemental analysis is for a desolvated version of **3** (% calcd. for C_1944_H_2976_B_6_Cr_126_F_168_N_36_Ni_18_O_600_Pd_6_, % found): C (48.66, 48.59), H (6.30, 6.14), Cr (13.66, 13.52), N (1.05, 1.00), Ni (2.20, 2.18), Pd (1.33, 1.32). The assembly does not give an interpretable NMR spectrum due to paramagnetism and slow tumbling.

### Crystallography data collection

X-ray data for **3** were collected on beamline i19 at Diamond Light Source equipped with a Saturn 724+ detector and an Oxford Cryosystems Cryostream 700 plus^61^. Data were processed and reduced using Rigaku Oxford Diffraction CrysAlisPro and absorption correction was performed using empirical methods (SCALE3 ABSPACK) based on symmetry-equivalent reflections combined with measurements at different azimuthal angles^62^. Crystals of the sample were very weakly diffracting and despite the use of synchrotron radiation, no reflections were observed beyond 1.5 Å resolution. The data were truncated accordingly.

### Crystal structure determinations and refinements

The structure was initially solved using a Patterson superposition using Shelxs and the model was refined using Shelxl from the initial solution^63^. After some refinements and with some features, including the thread and atoms coordinating to the metal sites, beginning to appear, the model was heavily restrained in order to ensure chemically sensible species were refined to the low-resolution electron density map.

Fixed distance restraints were applied to the majority of 1,2- and 1,3 distances of the thread, along with rigid body models for the phenyl and pyridyl rings and flat restraints for substituents to the phenyl and pyridyl rings. Hydrogens were placed in calculated positions.

The {Cr_7_Ni} rings were initially modelled as residues, made to be similar using a SAME restraint. Fixed distance restraints were applied to every 1,2- and 1,3 distances, along with flat restraints to the carboxylate groups. Hydrogens were placed in calculated positions. These were allowed to refine freely until convergence.

At this point a solvent mask was calculated using BYPASS through Olex2 and applied to the structure in order to account for diffuse electron density arising from disordered solvent and BF_4_ counter anions that could not be located in the low-resolution electron density map^64,65^. The structure was again refined until convergence.

The {Cr_7_Ni} residues were then fixed and each refined as rigid body model about a central dummy atom, in order to reduce the number of refinement parameters in order to maintain a reasonable data to parameter ratio. All restraints used are retained as comments in the embedded Shelx instruction file in the CIF for reference.

Similar neighbour atomic displacement parameter restraints were applied to the whole model. A BUMP restraint was also applied to the whole model to ensure no inappropriate short contacts. Non-metal atoms were left isotropic on account of the low number of data points due to the low resolution of the data-set.

2*F*_o_−*F*_c_ Electron density maps for the Supplementary Information Figures above were generated using the software packages PHENIX and COOT^66,67^.

CCDC 1860091 contains the Supplementary Crystallographic Data for this paper. These data can be obtained free of charge via www.ccdc.cam.ac.uk/conts/retrieving.html (or from the Cambridge Crystallographic Data Centre, 12 Union Road, Cambridge CB21EZ, UK; fax: (+44)1223-336-033; or deposit@ccdc.cam.ac.uk).

### Magnetic measurements

Variable-temperature (2.0–300 K) direct current (dc) magnetic susceptibility measurements under an applied field of 1000 G and variable-field (0–7.0 T) magnetization measurements at low temperatures (2.0 and 4 K) were carried out for **3** constrained in eicosane with a Quantum Design MPMS-XL7 SQUID magnetometer. The susceptibility data were corrected for the diamagnetism of the constituent atoms, the eicosane and the sample holder.

### EPR spectroscopy

Continuous wave X-band (ca. 9.5 GHz) EPR spectra of frozen solutions of **3** were recorded with a Bruker EMX580 spectrometer at the EPRSC National UK EPR Facility and Service at The University of Manchester. The data were collected at 5 K using liquid helium. Spectral simulations were performed using the EasySpin 4.5.5 simulation software^68^.

Pulsed EPR measurements were performed at low temperatures on a Bruker ElexSys E580 spectrometer operating at X-band frequency (ca. 9.5 GHz). 1 × 10^–4^ M toluene solutions of compound **3** were used (Supplementary Figs. 10–12, Supplementary Table 2).

### Inversion recovery

The inversion recovery pulse sequence used was *π*−*τ*−*π*/2−*τ*−*π*−*τ*−echo, with *π* = 32 ns, *τ* = 320 ns and variable *t*. The spin–lattice relaxation time constant, *T*_1_, was deducted by fitting the resulting signal to Eq. (1):

1 $$I\left( t \right) = I_1{\mathrm{exp}}\left( { - t/T_1} \right) + I_{{\mathrm{SD}}}\,{\mathrm{exp}}\left( { - t/T_{{\mathrm{SD}}}} \right),$$

where *I*_1_ and *I*_SD_ are the amplitudes and *T*_SD_ is the spectral diffusion time constant.

### Phase memory time, *T*_M_

The spin-echo decay measurements were carried out by gradually increasing the inter-pulse delay *τ* of a primary Hahn echo sequence *π*/2−*τ*−*π*−*τ*−echo. With microwave pulses of length *π* = 32 ns, strong proton–electron spin modulation was observed. In order to suppress such modulation, microwave pulses of length *π* = 128 ns were used. The phase memory time *T*_M_ could be deducted by fitting the experimental data to Eq. (2).

2 $$I(2{\mathrm{\tau }}) = I\left( 0 \right){\mathrm{exp}}\left[ {( - 2{\mathrm{\tau }}/T_{\mathrm{M}})^s} \right],$$

where *s* is a stretching parameter^69^.

### Solution-phase SAXS

SAXS measurements were performed on a HECUS SAXS/GISAXS instrument equipped with a XENOCS micro focus Cu*K*_α_ (*λ* = 1.5407 Å) source equipped with Montel optics and the diffracted X-rays collected with a Dectris Pilatus 100 K 2D detector. Sample **3** was dissolved in tetrahydrofuran contained in borosilicate capillaries with a diameter of 1 mm and a wall thickness of 10 μm. Silver behenate was used for calibration of the instrument before every collection. Pure tetrahydrofuran collection was performed with identical conditions as the samples to allow consistent subtraction. Sample collections typically took 10,000 s. All experimental data are the sum of the 2D radial distribution of the small-angle X-ray diffraction converted to a 1D line graph. Irena SAS/SANS routines in Wavemetrics Igor Pro have been used for calibration^70^, data conversion and subsequent analysis.

The analysis involved subtracting the solvent contribution from the sample + solvent data and then employing routines in Irena for the analysis. Pair distance distribution functions provided a reliable, simple and reproducible means for investigating the molecular sizes. The corrected data were analysed using the Moores method^70^. Initially the approximate size is determined and then function fitted to a region between large aggregate signals (small angles) and the statistically insignificant data at high angles. Fitting was repeated until a steady maximum size was achieved.

### Atomistic molecular dynamics simulations

We set up an all-atom simulation of the [13]rotaxane in THF solution using Gromacs 5.1.4 molecular dynamics package^50,51^. The initial model based on the crystal structure Cartesian coordinates for the [13]rotaxane was obtained experimentally and parameterized using the AMBER95^52^. Forcefield as implemented in GROMACS, augmented by parameters consistent with the General Amber Forcefield^52^. Additional harmonic restraints were added to maintain the integrity of the Pd cage and [Cr_7_NiF_8_(O_2_C^*t*^Bu)_16_] rings during the simulation. A single crystal structure in a solution of 9356 THF molecules at a density of 890 kg/m^3^ in a cubic box size of 11.00 nm^3^ with 6 BF_4_^−^ ions to neutralize the system was set up.

The system was minimized with the conjugate gradient algorithm prior to the simulation runs. The minimized structure was run for 10 ns using an NPT ensemble (*T* = 298 K and *p* = 1.01325 bar) using the Nosé–Hoover thermostat and the Parrinello–Rahman barostat. Periodic boundary conditions were used and the time step for integration was fixed at 1 fs with the neighbour list updated every 10 fs. The particle-mesh Ewald method was used to evaluate the electrostatics.

SAXS was calculated based on the crystal structure set up for Molecular Dynamics simulation to be compared directly to SAXS data from experiments. To simulate the experimental subtraction of solvent, the calculated SAXS also excludes the effect of background solvent. We employed the approach implemented in GROMACS, which directly computes the scattering intensity from the scattering vectors and the scattering factors. Scattering factors for the metal ions were taken from computed X-ray scattering factors from Hartree–Fock calculations^71^. The SAXS box used for all calculated profiles was 100 nm with a X-ray wavelength of 0.154209 nm. SAXS profiles were plotted as a natural log of intensity ln *I*(*q*)(a.u.) against the scattering vector, *q* (Å). SAXS calculation were done on the whole structure and various structural variations (see Supplementary Table 3).

Irena SAS routines in Igor Pro have been used to calculate the pair distribution function from the SAXS data obtained from Gromacs^51^. The Pair distribution function using routines in Irena has enables us to investigate and compare the molecular size to experimental data. Moores method^71^ was used to approximate the size and then fitted to a function in the region of large aggregate signals (small angles) and the statistically insignificant data at high angles. The fitting was repeated for all data sets until a maximum size was achieved.

The SAXS profile and *P*(*r*) functions were all visually inspected and compared directly to corresponding experimental data.

## Supplementary information

Supplementary Information

## Data Availability

Supplementary information is available in the online version of the paper. CCDC 1860091 contains the supplementary crystallographic data for this paper. These data can be obtained free of charge via www.ccdc.cam.ac.uk/conts/retrieving.html (or from the Cambridge Crystallographic Data Centre, 12 Union Road, Cambridge CB21EZ, UK; fax: (+44)1223-336-033; or deposit@ccdc.cam.ac.uk). Reprints and permissions information is available online at www.nature.com/reprints. Correspondence and requests for materials should be addressed to R.E.P.W.
