# Supplementary material for: A [13]rotaxane assembled via a palladium molecular capsule
Source: Nat Commun. 2019 Aug 16;10:3720. doi: 10.1038/s41467-019-11635-6 (PMC6697691; doi:10.1038/s41467-019-11635-6)
Supplement: Supplementary file 1 — Supplementary Information [file 41467_2019_11635_MOESM1_ESM.docx]

**Supplementary Figures**


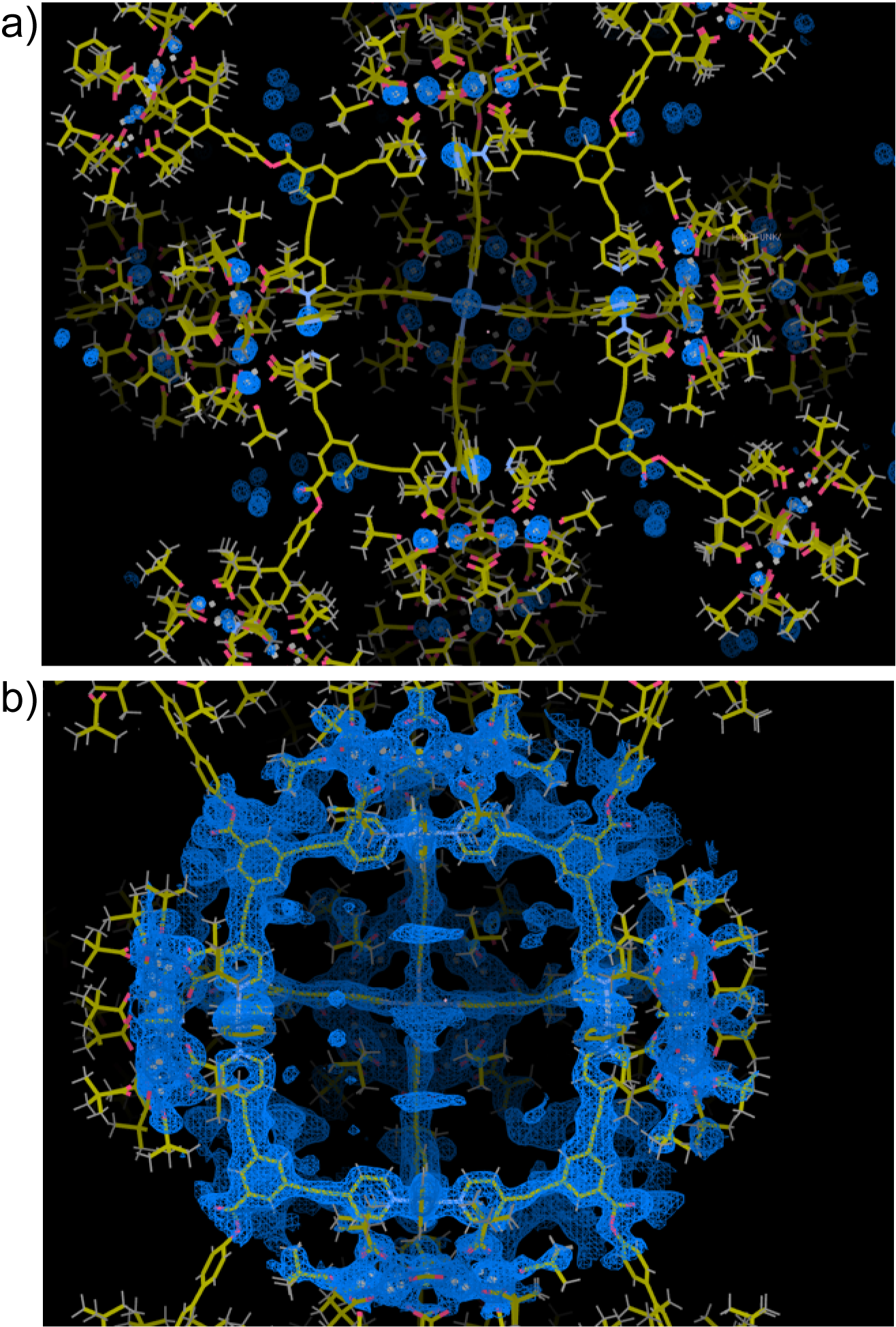


**Supplementary Figure 1.** (a) 2F_o_ – F_c_ Electron density map and model representation scaled to show only the metal sites (map level 8.31 e/Å^3^); (b) the same projection scaled to show the thread and {Cr_7_Ni} ligands (map level 2.48 e/Å^3^).


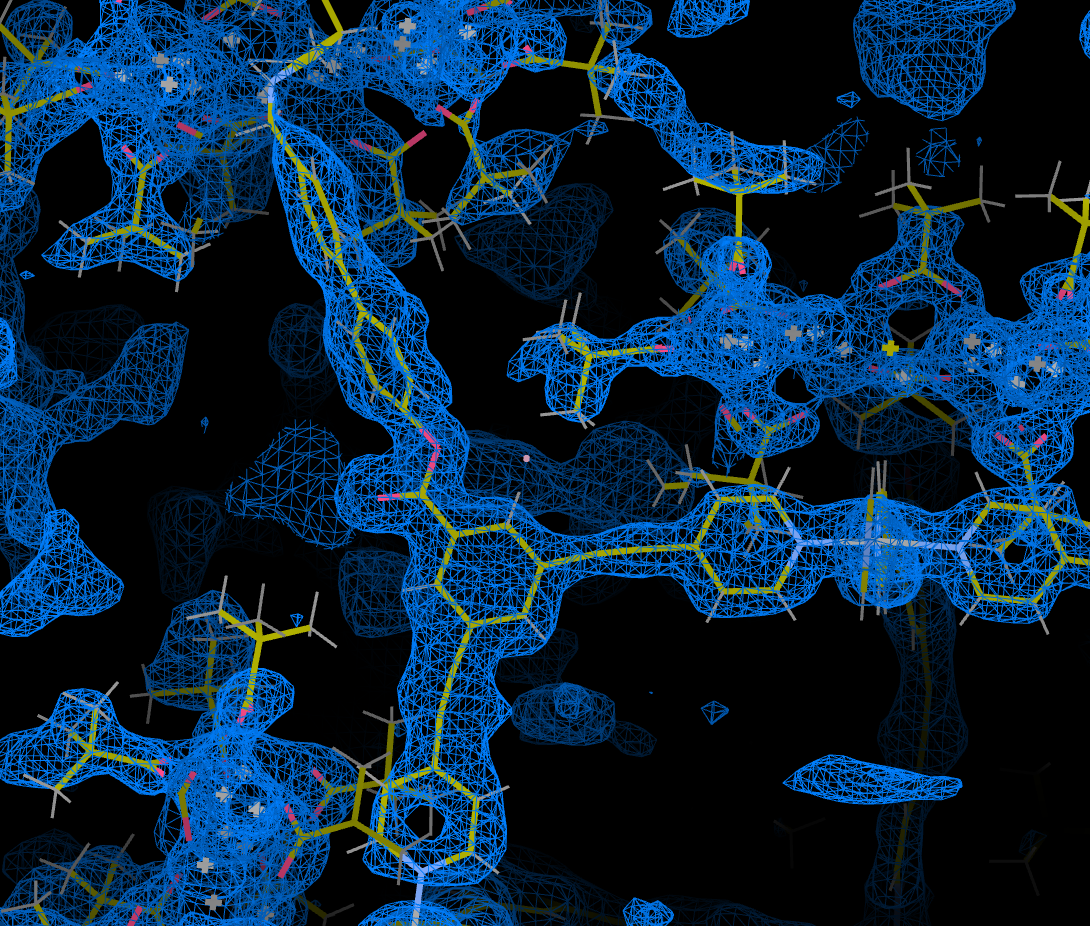


**Supplementary Figure 2**. 2F_o_ – F_c_ Electron density map and model representation showing a close up of the thread (map level 2.48 e/Å^3^).


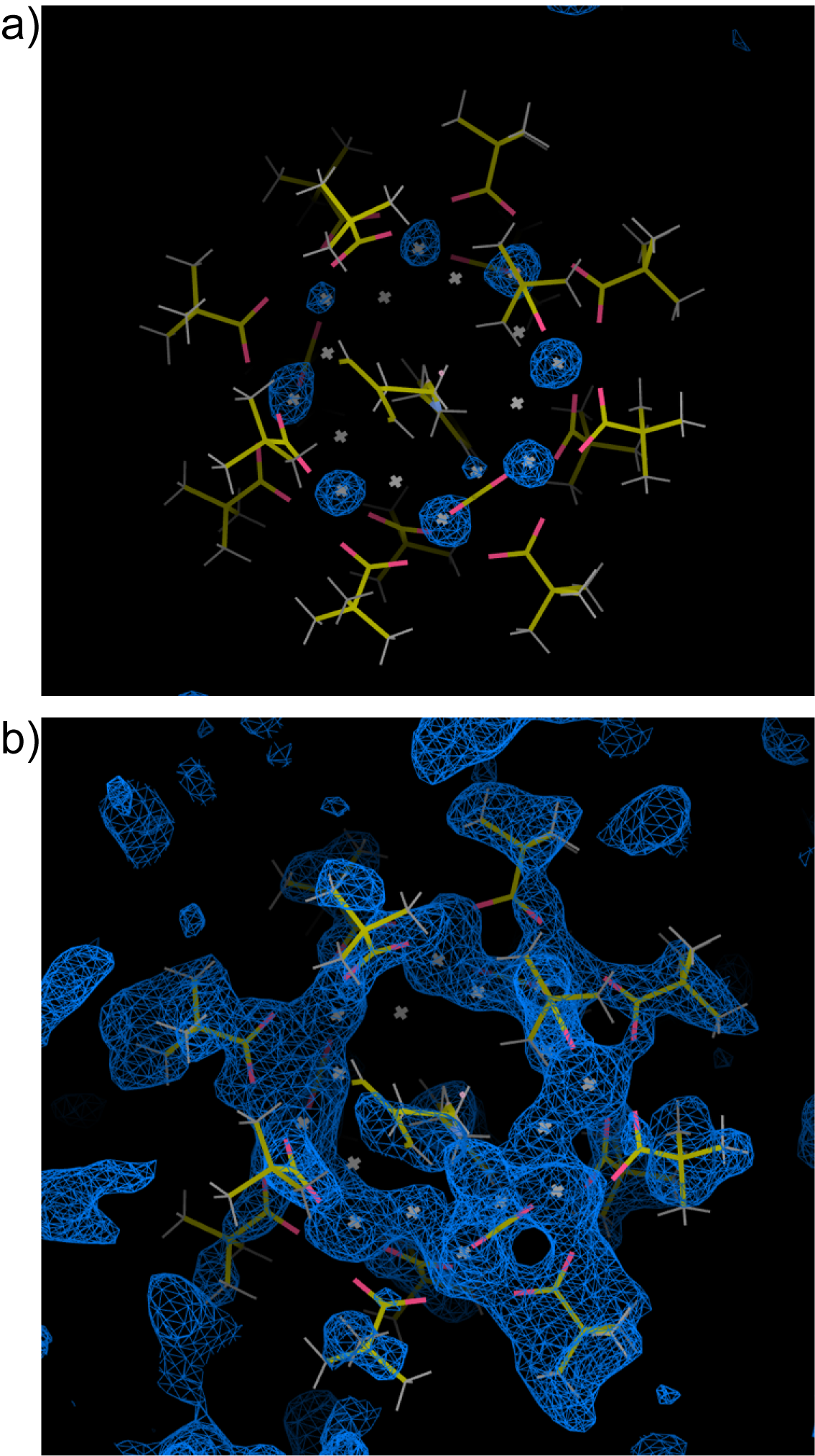


**Supplementary Figure 3**. 2F_o_ – F_c_ Electron density map and model representation showing one of the peripheral rings (ring 4) (a) scaled to show only the metal sites (map level 5.31 e/Å^3^) and (b) scaled to show the ligands and thread (map level 1.95 e/Å^3^).


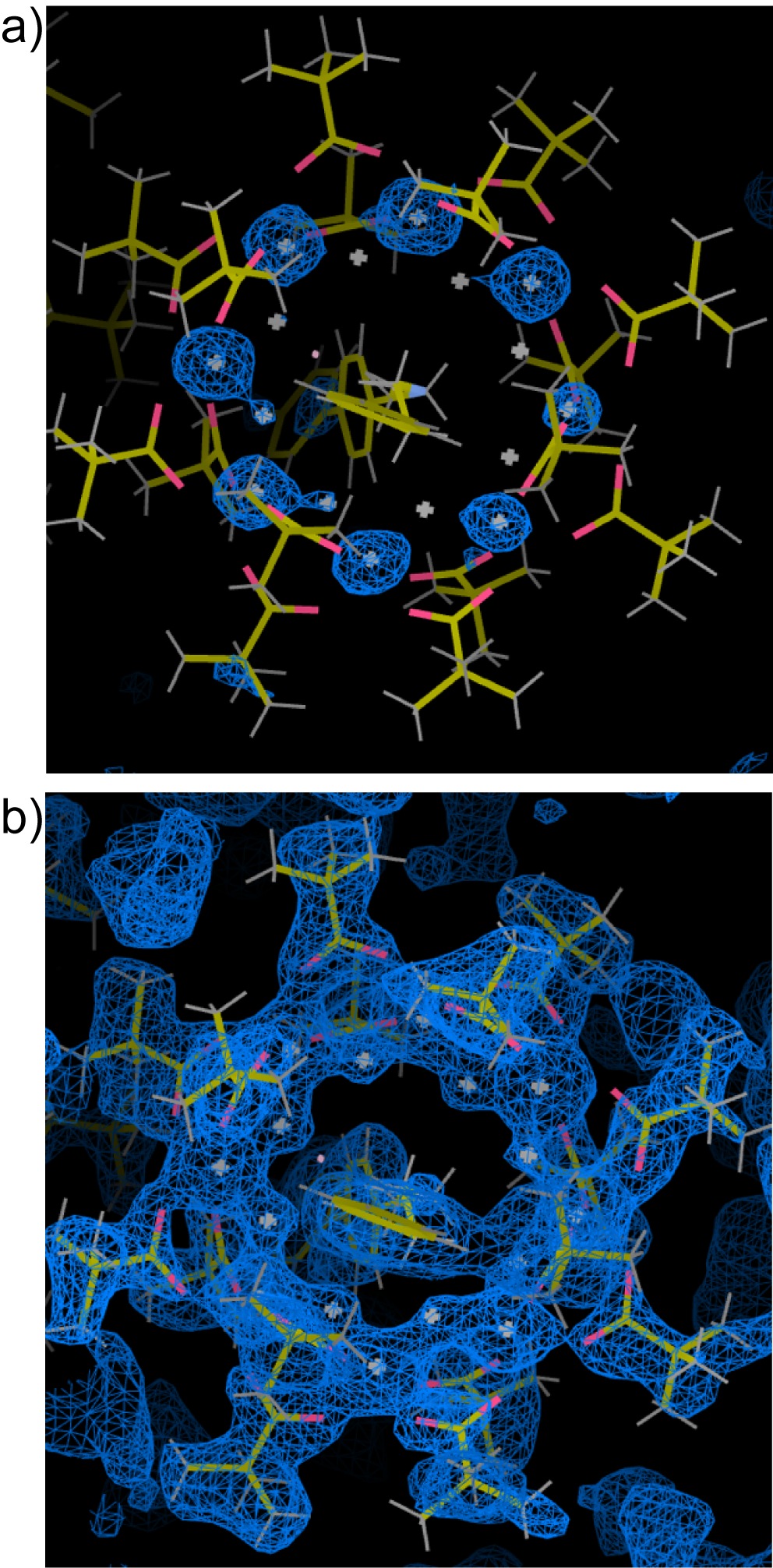


**Supplementary Figure 4**. 2F_o_ – F_c_ Electron density map and model representation showing one of the peripheral rings (ring 3) (a) scaled to show only the metal sites (map level 5.31 e/Å^3^) and (b) scaled to show the ligands and thread (map level 1.95 e/Å^3^).

**
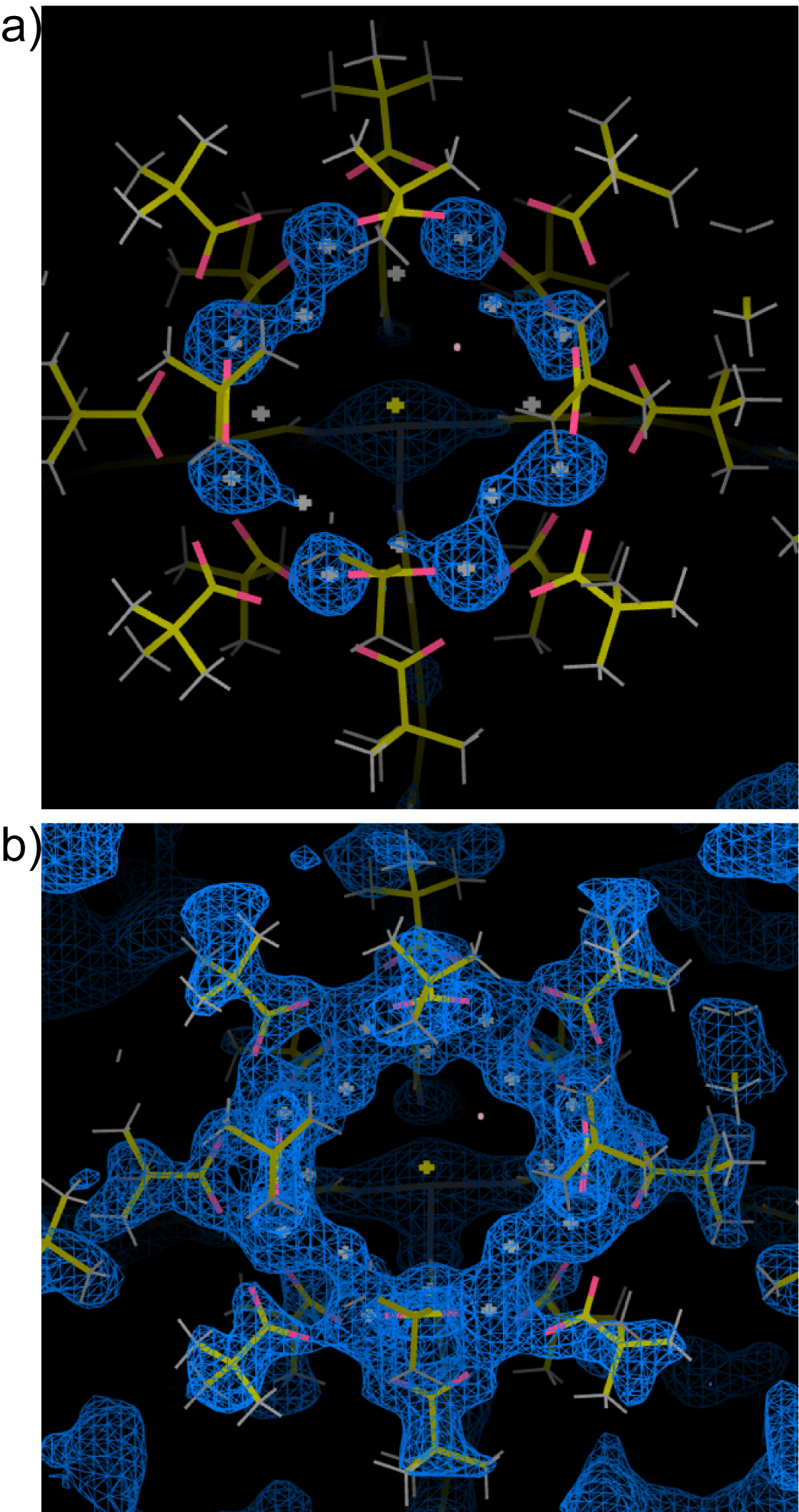
**

**Supplementary Figure 5**. 2F_o_ – F_c_ Electron density map and model representation showing one of the charge-balancing rings (ring 2) (a) scaled to show only the metal sites (map level 5.31 e/Å^3^) and (b) scaled to show the ligands and thread (map level 1.95 e/Å^3^).


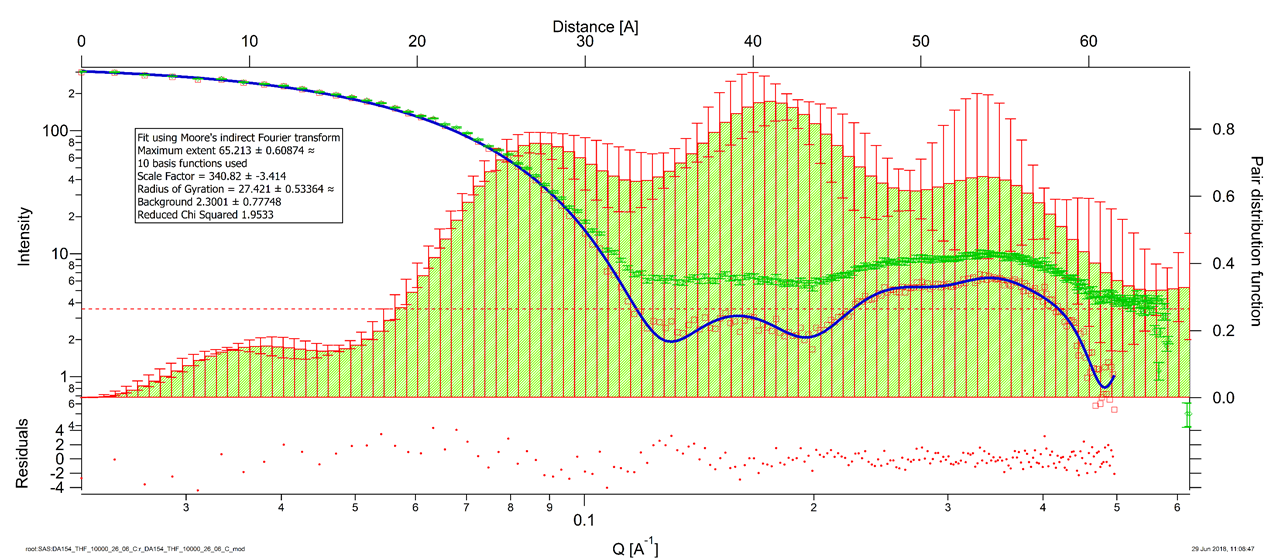


Q (A^-1^)

**Supplementary Figure 6.** Experimental SAXS data of compound **3** shown in green crosses. Green columns with error bars is the pair distribution function that is associated with the fit of the data shown in solid blue line.

**
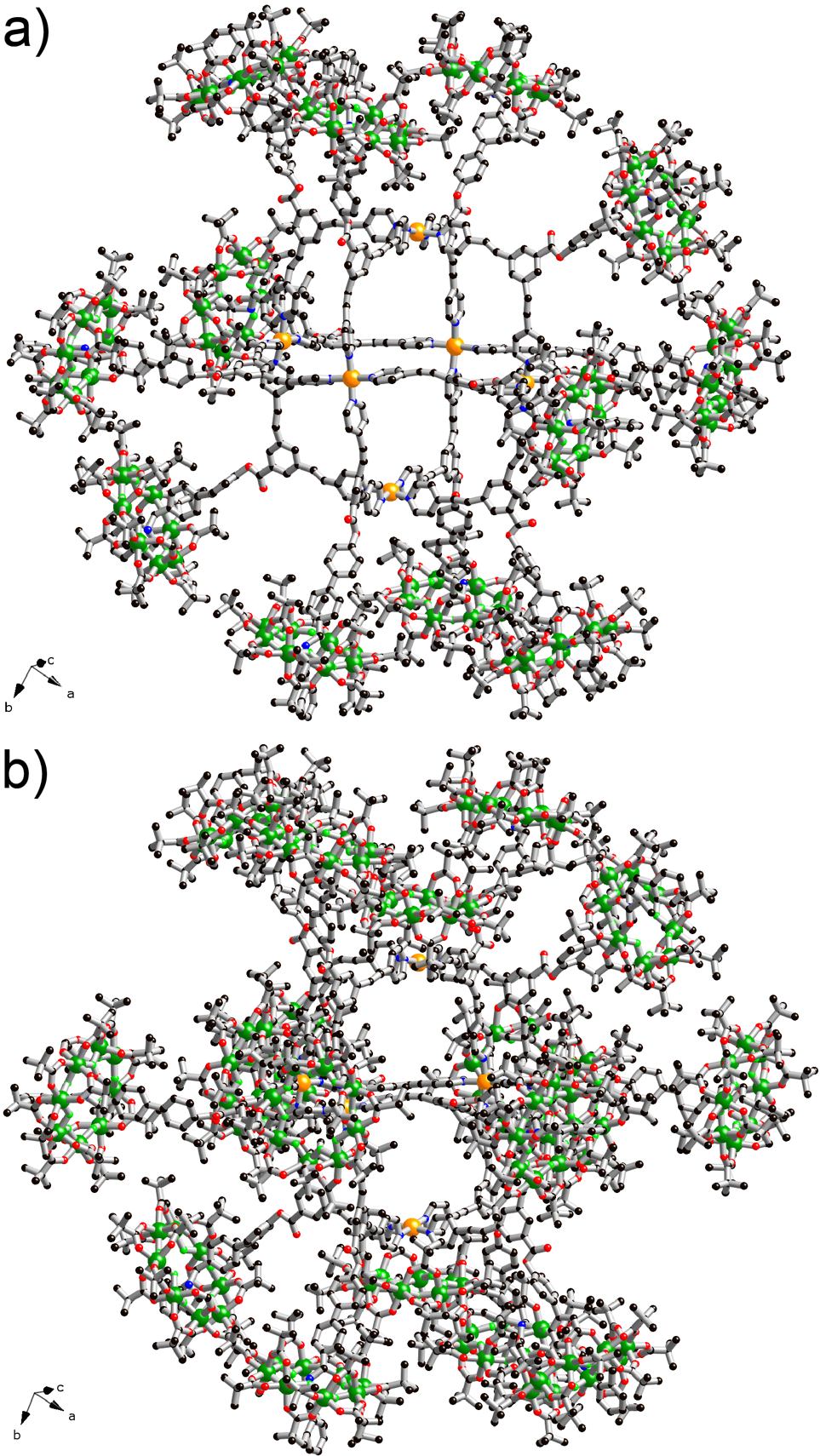
**

**Supplementary Figure 7.** Perspective views of the [13]rotaxane in **3** without (a) and with [Cr_7_Ni(*µ*-F)_8_(O_2_C*^t^*Bu)_16_]^–^ counteranions (b). Colour code: Cr, green; Ni, purple; N, blue; O, red; C, grey; F, pale green; Pd, tangerine. H atoms omitted for clarity.

**
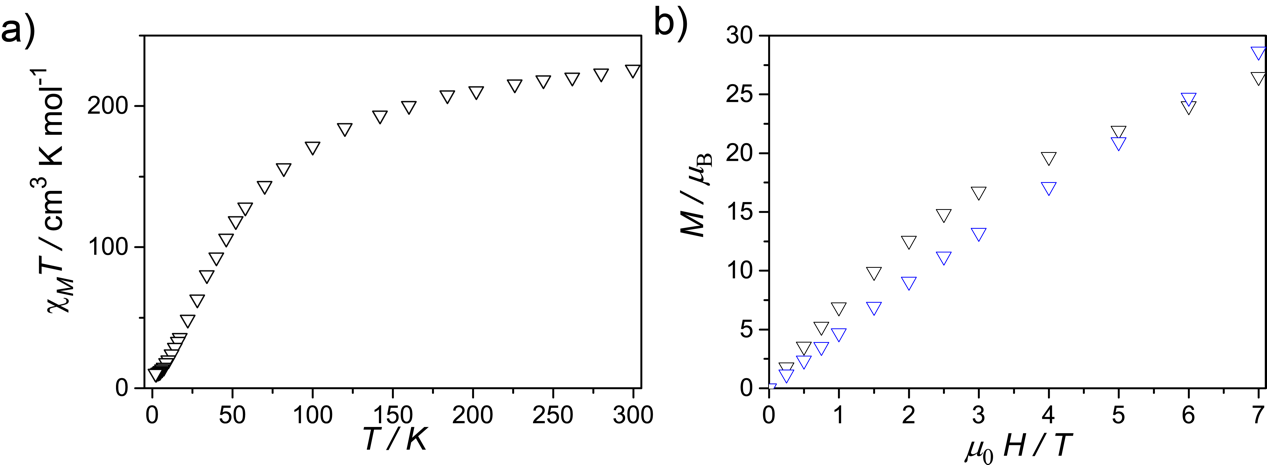
**

**Supplementary Figure 8.** Temperature dependence of the product of the direct current (dc) molar magnetic susceptibility by the temperature (*χ*_M_*T*) (a) and the magnetic field dependence of the molar magnetisation (*M*) at 2 (▽) and 4 (▽) K (b) for **3**.

**
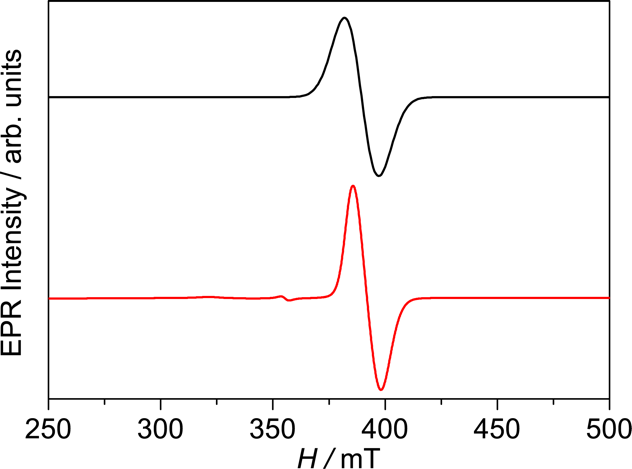
**

**Supplementary Figure 9.** Experimental (black trace) and simulated (red trace) X-band continuous wave electron paramagnetic resonance on frozen solutions of **3** at 5K. {Cr_7_Ni} ring *S* = ^1^/_2_; *g_x_* = 1.78; *g_y_* = 1.78; *g_z_* = 1.74.

**
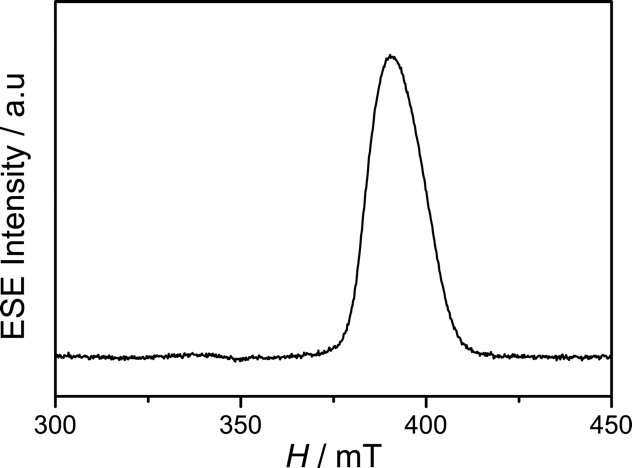
**

**Supplementary Figure 10.** Echo-detected field-swept spectra at X-band for **3** in 1 x 10^-4^ M toluene solutions, recorded with a primary echo sequence π/2-τ-π-τ-*echo* with π = 32 ns and *τ* = 300 ns.

**
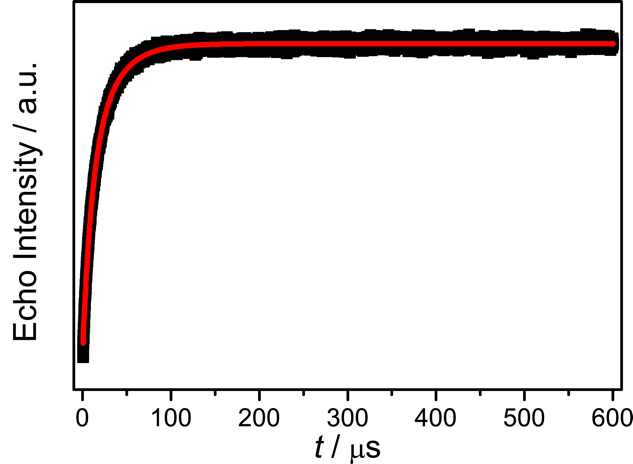
**

**Supplementary Figure 11.** Electron-spin-echo (ESE) inversion recovery curves for **3** at X-band in diluted (1 x 10^-4^ M) toluene solutions at the maximum resonance field for the ring. The red line is a fit to the exponential decay function *I*(t) = *I*_1_ exp(-*t*/*T*_1_) + *I*_SD_ exp(-*t*/*T*_SD_) from which the time constants *T*_1_ and *T*_SD_ were deducted (see Table S2).

**
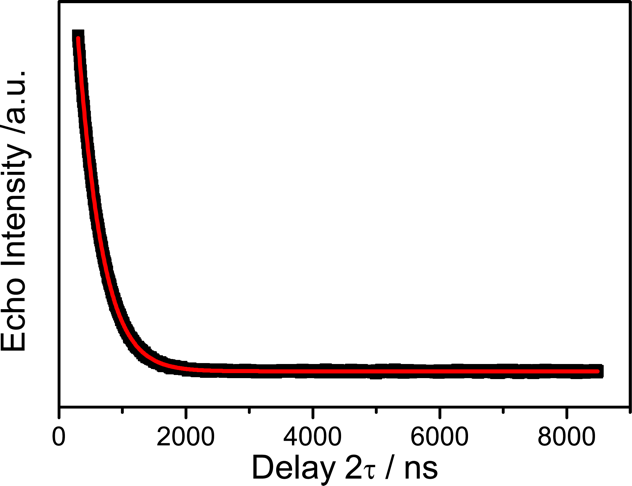
**

**Supplementary Figure 12.** Two pulse electron spin-echo decays at X-band for **3** in diluted (1 x 10^-4^ M) toluene solutions at the maximum resonance field for the ring, recorded with pulses of π = 128 ns. The red lines represent fits to Eq. 1, *I*(2*τ*) = *I*(0)exp[(-2*τ*/*T_M_*)^s^], with *T*_M_ and *s* values listed in Table S2.

**Supplementary Tables**

**Supplementary Table 1.** Crystallographic information for compound **3**.

| Identification code | jsf353_2 |
| --- | --- |
| Empirical formula | C_1948_H_2982_Cr_126_F_144_N_38_Ni_18_O_600_Pd_6_ |
| Formula weight | 47516.70 |
| Temperature/K | 100.0(2) |
| Crystal system | tetragonal |
| Space group | I4 |
| a/Å | 53.0572(19) |
| b/Å | 53.0572(19) |
| c/Å | 56.013(4) |
| α/° | 90 |
| β/° | 90 |
| γ/° | 90 |
| Volume/Å^3^ | 157680(16) |
| Z | 2 |
| ρ_calc_g/cm^3^ | 1.001 |
| μ/mm^‑1^ | 0.563 |
| F(000) | 49672.0 |
| Crystal size/mm^3^ | 0.5 × 0.4 × 0.3 |
| Radiation | synchrotron (λ = 0.6889) |
| 2Θ range for data collection/° | 3.148 to 26.548 |
| Index ranges | -27 ≤ h ≤ 35, -34 ≤ k ≤ 35, -37 ≤ l ≤ 35 |
| Reflections collected | 80483 |
| Independent reflections | 23527 [R_int_ = 0.0810, R_sigma_ = 0.0676] |
| Data/restraints/parameters | 23527/3748/1351 |
| Goodness-of-fit on F^2^ | 1.089^c^ |
| Final R indexes [I>=2σ (I)] | R_1_ = 0.1341^a^, wR_2_ = 0.3339^b^ |
| Final R indexes [all data] | R_1_ = 0.1820, wR_2_ = 0.3720 |
| Largest diff. peak/hole / e Å^-3^ | 1.08/-0.38 |

^a^ *R1*(*F*) = Σ(|*F_o_*| – |*F_c_*|)/Σ|*F_o_*|; [b] *wR*^2^(*F*^2^) = [Σ*w*(*F_o_*^2^ – *F_c_*^2^)^2^/Σ*wF_o_*^4^]^½^; [c] *S*(*F*^2^) = [Σ*w*(*F_o_*^2^ – *F_c_*^2^)^2^/(*n* + *r* – *p*)]^½^

**Supplementary Table 2.** Selected X-band pulsed-EPR^a^ data for **3**.

| Compound | *T* / K | *T*_1_ / ns | *T*_SD_ / ns | *T*_M_ / ns | *s* |
| --- | --- | --- | --- | --- | --- |
|  |  |  |  |  |  |
| **3** | 2.5 | 26958 ± 312 | 9606 ± 191 | 425 ± 2 | 1.11 ± 0.004 |
|  |  |  |  |  |  |
| *^a^*Measurement performed on a Bruker ElexSys E580 spectrometer operating at X-band frequency (*ca.* 9.5 GHz), in diluted (0.0001 M) toluene solution at the maximum resonance field for the ring in each compound. *T*_1_ represents the spin-lattice relaxation time constant and *T*_SD_ the spectral diffusion time constant. | | | | | |

**Supplementary Table 3.** Structures used in atomistic molecular dynamics simulations.

| **Name** | **Structures** | **No of Ligand Rings** | **No of anion rings** | **Total No of atoms*** | **Rg (Å)** |
| --- | --- | --- | --- | --- | --- |
| **Whole structure**  **(Fig 2b)** | **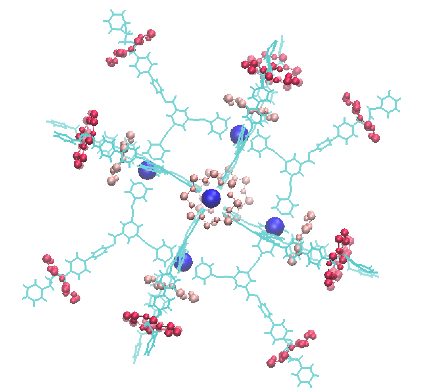** | **12** | **6** | **5850** | **27.0±0.0116** |
| **No anion rings**  **(Fig 2a)** | **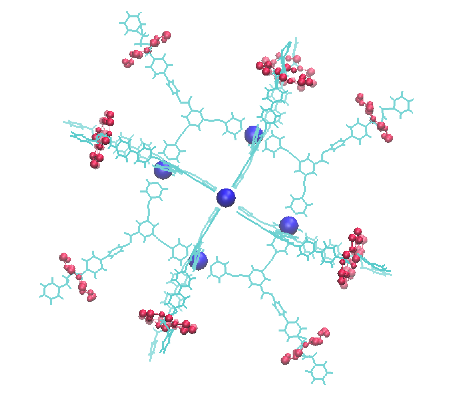** | **12** | **0** | **4218** | **21.9±0.0198** |
| **No rings within the rotaxanes** | **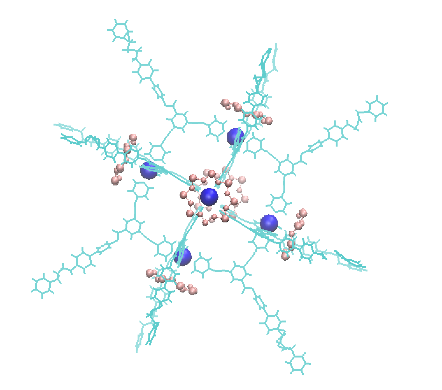** | **0** | **6** | **2586** | **18.6±0.0224** |
| **Half rings within rotaxanes** | **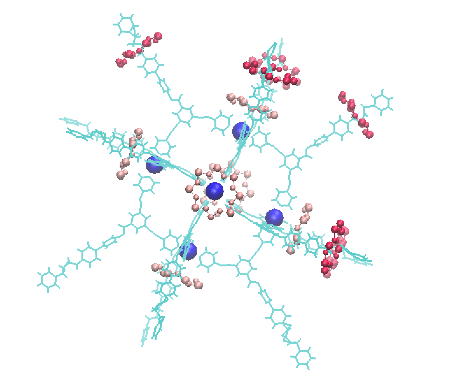** | **6** | **6** | **2586** | **24.6±0.0249** |
| **Core with no rings** | **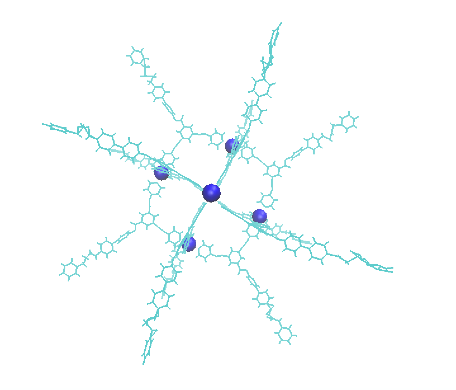** | **0** | **0** | **954** | **17.0±0.0247** |

*Total number of atoms excluding solvent.
